# The Destiny of Glucose from a MicroRNA Perspective

**DOI:** 10.3389/fendo.2018.00046

**Published:** 2018-02-26

**Authors:** Paola Mirra, Cecilia Nigro, Immacolata Prevenzano, Alessia Leone, Gregory Alexander Raciti, Pietro Formisano, Francesco Beguinot, Claudia Miele

**Affiliations:** ^1^Istituto per l’Endocrinologia e l’Oncologia Sperimentale “Gaetano Salvatore” - CNR, Naples, Italy; ^2^Department of Translational Medical Sciences, University of Naples Federico II, Naples, Italy

**Keywords:** glucose metabolism, insulin signaling, insulin resistance, microRNAs, posttranscriptional regulation

## Abstract

Glucose serves as a primary, and for some tissues the unique, fuel source in order to generate and maintain the biological functions. Hyperglycemia is a hallmark of type 2 diabetes and is the direct consequence of perturbations in the glucose homeostasis. Insulin resistance, referred to as a reduced response of target tissues to the hormone, contributes to the development of hyperglycemia. The molecular mechanisms responsible for the altered glucose homeostasis are numerous and not completely understood. MicroRNAs (miRNAs) are now recognized as regulators of the lipid and glucose metabolism and are involved in the onset of metabolic diseases. Indeed, these small non-coding RNA molecules operate in the RNA silencing and posttranscriptional regulation of gene expression and may modulate the levels of kinases and enzymes in the glucose metabolism. Therefore, a better characterization of the function of miRNAs and a deeper understanding of their role in disease may represent a fundamental step toward innovative treatments addressing the causes, not only the symptoms, of hyperglycemia, using approaches aimed at restoring either miRNAs or their specific targets. In this review, we outline the current understanding regarding the impact of miRNAs in the glucose metabolism and highlight the need for further research focused on altered key kinases and enzymes in metabolic diseases.

The glucose metabolism provides the principal source of cellular energy and substrate storage. Indeed, although several other components can be used for fuel by muscles and other tissues in the fasted state, a continuous supply of glucose is necessary as a source of energy, especially for the cells of the nervous system and the erythrocytes, which have little or no capability of storing glucose and using other substrates as fuel. The maintenance of a normal glucose balance or homeostasis depends on several simultaneous ongoing processes in different tissues, including the liver, skeletal muscle, and adipose tissue, and it is under the control of insulin. Indeed, insulin is considered the principal hormone in the glucose homeostasis, suppressing hepatic gluconeogenesis and promoting glycogen synthesis and storage in the liver and muscle, triglyceride storage in the adipose tissue and amino acid storage in the muscle. Thus, in insulin-resistant conditions, a decrease in the blood glucose clearance occurs, while the hepatic glucose output and release in the plasma persist, contributing to the hyperglycemia, namely high glucose levels in the bloodstream (1). Un-treated chronic hyperglycemia is responsible for long-term complications.

The insulin signal transduction machinery has been explained in detail over the past few decades and the existence of molecular defects in the pathways activated by insulin, which contribute to metabolic disorders, are undeniable (2). However, the etiology of type 2 diabetes (T2D) is polygenic and heterogeneous, combining both genetic and environmental factors (3–5). Moreover, considering the delay between the onset and the diagnosis of T2D, which is often made clinically when complications are already manifest, it becomes necessary to engage in further investigations to establish other main causes of the disease for an early and more accurate prediction of high-risk individuals and for the identification of new therapeutic targets. From this perspective, in addition to the insulin signaling, it is crucial to consider also metabolic pathways in this search, examining key metabolic regulators and coordinators that may have a role in the onset of hyperglycemia.

To date, most studies have been concentrated on the transcriptional regulation mechanisms or posttranslational modifications of insulin signaling components or metabolic enzymes. By contrast, a conceptual gap exists regarding the role of posttranscriptional gene silencing by microRNAs (miRNAs) in the control of the energy metabolism in healthy as well as in obese and T2D individuals. The pressure to close this gap is due to mounting evidence that suggests a prominent role for miRNAs in the metabolism and metabolic disorders (6), in addition to the challenge currently facing scientists in the proposal of new therapeutic targets against hyperglycemia and T2D.

The purposes of this review are as follows:

– to highlight the recent progress in the study of miRNAs involved in the metabolism, with a special emphasis on metabolic abnormalities in insulin-resistant conditions;
– to report persistent limitations in the study of miRNAs involved in the glucose metabolism.

## Introduction on miRNAs

MicroRNAs are small (20–22 nucleotides), non-coding RNAs, characterized by an evolutionary conservation and able to regulate gene expression at the posttranscriptional level. In more detail, the miRNA seed region interacts with the complementary sequence in the 3′ untranslated region (UTR) of target messenger RNAs (mRNAs) and, subsequently, inhibits the translation or targets the degradation of the bound mRNAs (7). The miRNA biogenesis includes several steps: transcription, primary transcript (pri-miRNA) processing, transport to the cytoplasm, precursor miRNA (pre-miRNA) processing, and strand selection (8). This rigorous, multi-step process ensures that only those miRNAs with the correct structure and sequence are functional (9, 10).

Regarding transcription, miRNA genes are found in every chromosome, and miRNA promoters, like protein-coding genes, are regulated by epigenetic mechanisms and transcription factors. The complete definition of the gene structure is ongoing for all the identified miRNAs, as the genomic location and the gene-neighborhood context may be informative about specific miRNAs and miRNAs in general. miRNA genes are transcribed by RNA polymerase II or III from independent miRNA genes (intergenic) or from the exons or introns in transcription units of protein-coding or non-coding genes (intragenic). The intronic miRNAs are more common and better studied, showing a coordinated expression with the host gene. miRNA genes are frequently clustered in the genome and simultaneously transcribed into single poly-cistronic transcripts to realize an intricate regulatory network, being expressed at similar levels and in a coordinated manner. The intriguing aspect of the miRNA clusters is that they frequently contain representatives from different miRNA families, meaning that the members of a given cluster are able to regulate multiple different mRNAs. This feature is important because it implies that the regulation by miRNAs is extremely complex, involving several signaling pathways and gene networks. However, the transcriptional regulation of miRNAs, which represents the critical step in modulating their expression, remains poorly understood. This is due to limitations in the methodology available to study pri-miRNAs and in the characterization of their promoter regions. Therefore, it is necessary to identify the factors that control the transcription of specific miRNAs to suggest novel therapeutic strategies aimed at restoring the aberrant miRNA expression rather than treating their consequences.

The nascent transcript, referred to as the pri-miRNA, is usually several hundred nucleotides in length and contains one or more hairpin structures. The miRNA-specific processing begins in the nucleus with the cleavage at the stem-loop formations by an RNase III-like enzyme, known as Drosha, which acts in concert with co-factors, including DiGeorge syndrome critical region 8 (also known as Pasha in flies and nematodes), at the level of the Microprocessor complex. The result is a 70-nucleotide long stem-loop precursor, known as the pre-miRNA, which is then exported to the cytosol by exportin 5 and Ran-GTP through nuclear pores. Once in the cytoplasm, Dicer, another RNase III-like endonuclease, recognizes the hairpin-shaped pre-miRNA hairpin and cuts the terminal-loop to generate a RNA duplex, 20–22 nucleotides in length. To stabilize the interaction of Dicer with the pre-miRNA, the Dicer cleavage takes place in a large complex, which includes TRBP or TARBP2 (the human immunodeficiency virus trans-activating response RNA-binding protein).

The resulting miRNA duplex is composed of the mature miRNA and the base-paired passenger strand, sometimes indicated as the miRNA* strand. The final step consists in the incorporation of the miRNA duplex into the final effector complex, namely the RNA-induced silencing complex (RISC), where it is loaded onto an Argonaute (Ago) protein. At this point, the strand that exhibits less stable base pairing at the 5′ end remains associated with the Ago protein (mature miRNA), while the other is unwound from the duplex and typically degraded by the RISC (miRNA* strand), although some miRNAs* are thought to regulate gene expression like mature miRNAs. However, it is not known why some miRNAs* are functional and others are not. One hypothesis is that the two strands are used differently in response to extracellular or intracellular signals, regulating diverse sets of protein-coding genes as needed; an alternative hypothesis is that the working strand could be selected in a tissue-specific manner (10–17).

It has been estimated that approximately 60% of protein-coding genes are influenced by miRNAs (18). In addition, recent advances in bioinformatics and biochemical methods for the study of miRNAs are currently allowing the identification and validation of a growing number of targets, strengthening the functions of miRNAs (19, 20). Indeed, they are involved in many key biological processes. As a consequence, their aberrant expression may be responsible for several pathophysiological conditions, suggesting that miRNAs could serve as novel targets for preventive or therapeutic interventions. However, since a single miRNA has rather a modest effect on the protein levels of specific targets, multiple concurrent actions on functionally related targets are necessary to observe a noteworthy effect due to this specific miRNA, which is possible as it targets hundreds of genes. In addition, multiple miRNAs may act in concert to exert a more potent additive effect on one specific mRNA target, as multiple miRNA binding sites are predicted in its 3′ UTR.

## The Relevance of miRNAs in the Metabolism

In the past few decades, miRNAs have emerged as key regulators of different aspects in development, homeostasis, and function (21, 22). In addition, their importance in the control of the metabolism has also been revealed, as miRNA research in the metabolic field has progressed rapidly in the recent years (23).

At the present time, however, only a handful of miRNAs have been identified as capable of mediating adipocyte differentiation and function (24, 25), controlling β cell mass and insulin secretion (26, 27) and co-targeting multiple nodes in the insulin signaling pathway (28) at the level of the skeletal muscle, adipose tissue, and liver (29). Moreover, for the identified miRNAs, only a few important targets have been clearly defined, while most miRNA targeting remains undisclosed.

Nevertheless, it is now indisputable that an aberrant expression and function of miRNAs contribute to the loss of the glucose homeostasis, leading to pathogenic conditions (30, 31). Therefore, to assess the involvement of miRNAs in T2D, most research efforts have addressed the expression profile of miRNAs mainly in animal models and, to a lesser extent, in diabetic patients. Although changes in miRNA expression levels have been demonstrated in humans, it is not possible to gain a clear picture at the moment, considering the limited available data and important drawbacks, such as the small cohort size and confounding factors related to age, body mass index, and sex differences, in addition to the lack of uniformity in miRNA measurement techniques (32).

Several promising studies have demonstrated that the manipulation of specific miRNAs *in vivo*, mostly in the liver, can modulate metabolic phenotypes and even reverse the course of insulin resistance and diabetes (33), suggesting that miRNA-based approaches may represent a viable strategy for treating metabolic diseases in the future (34, 35). However, more knowledge is needed on current obstacles ranging from inadequate delivery strategies to possible side effects.

Furthermore, it has been demonstrated that circulating miRNAs are detectable and stable in body fluids and that altered circulating miRNA profiles are associated with metabolic diseases, including T2D (36). For this reason, circulating miRNAs show great promise as novel biomarkers for such disorders and/or their associated complications. This aspect will be especially useful in the case of T2D (37), which is currently screened for and diagnosed by reliable biological tests based on blood glucose levels, but lacks of the means to detect at-risk patients or to monitor diabetic complications. Therefore, the availability of easily detectable biomarkers able to identify subjects who are in the early, pre-clinical stages of T2D or who are destined to suffer from it, in addition to monitoring the progression of diabetic complications and the response to treatments, may allow the implementation of personalized therapies. However, the studies performed to date are insufficient to warrant application of circulating miRNAs in clinical practice, proving to be highly heterogeneous in size and inclusion criteria, and not replicable. Hence, several key issues need to be addressed, before they can be used as biomarkers. A major challenge is to establish robust protocols for pre-analytical sample handling, miRNA extraction, and measurement, which are all important for the reproducibility of the results. Additionally, planning studies need to be conducted using well-characterized and larger cohorts to gain sufficient statistical power. Finally, sets of miRNAs characterizing the various metabolic diseases are more preferable rather than single miRNAs to be used as biomarkers to increase the specificity of the analysis.

## The Direct and Indirect Effects of miRNAs on the Insulin Signaling Pathway

Insulin, interacting with a specific tyrosine kinase receptor, propagates signals *via* two main branches: the phosphatidylinositol 3-kinase (PI3K)–AKT/protein kinase B (PKB) pathway, which is responsible for most of the metabolic effects of the hormone, and the RAS-mitogen-activated protein kinase pathway, which regulates gene expression and controls proliferation, differentiation, and survival at the cellular level. Therefore, the insulin signal transduction must be tightly controlled to avoid severe metabolic and proliferative perturbations. To ensure a proper signal duration and intensity, negative regulators depend on insulin itself. These feedback mechanisms, based on the action of phosphatases or inhibitory proteins, turn off the insulin signal in a rapid manner at different, essential sites. Among the hundreds of molecules involved in the insulin signaling pathway, the insulin receptor (IR)/insulin receptor substrate protein (IRS) interaction, the PI3K heterodimer and AKT/PKB have been identified as critical nodes (38). However, some of the inhibitory mechanisms on these nodes can be altered in pathophysiological conditions, participating in the development of insulin resistance and, in turn, contributing to the metabolic abnormalities in insulin-sensitive cell types, namely adipocytes, hepatocytes, and myocytes (2). A large number of studies have demonstrated defects in a myriad of processes, often concomitantly, in various tissues and cell types. Nevertheless, as the causes of the reduced responsiveness to insulin are numerous and multifactorial, deciphering the complexity of the insulin resistance pathogenesis remains one of the great challenges on the way toward developing new molecules for a more effective treatment of T2D and associated diseases.

**Figure 1:**
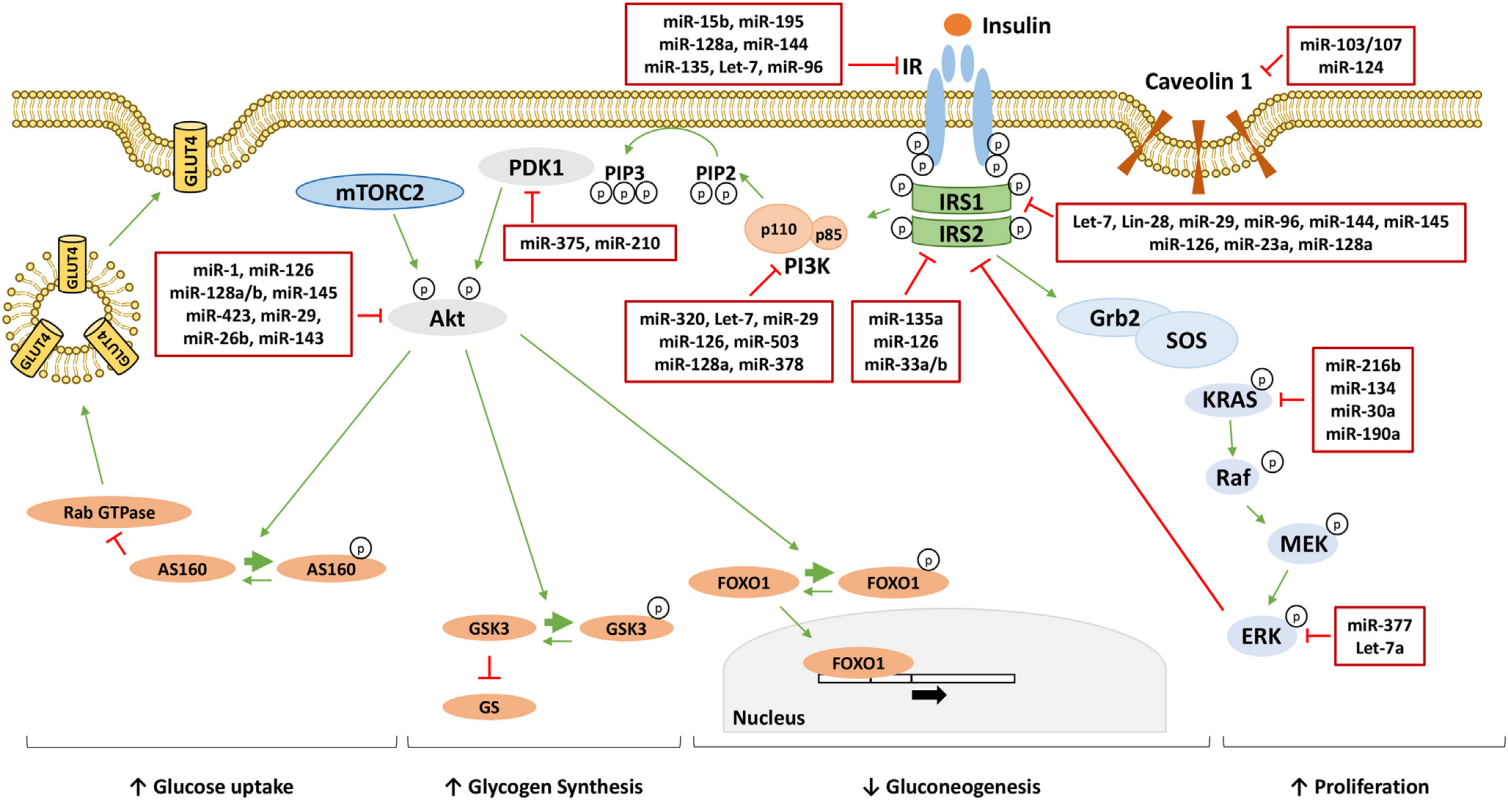
MicroRNAs (miRNAs) targeting insulin signaling mediators. Following the binding to insulin receptor (IR), insulin induces the activation of phosphatidylinositol 3-kinase (PI3K)/AKT pathway responsible for the metabolic effect of insulin through the increase of glucose uptake, the increase of glycogen synthesis and the reduction of gluconeogenesis. On the other hand, insulin activates the mitogen-activated protein kinase (MAPK) pathway increasing cellular proliferation. miRNAs directly targeting critical nodes of insulin signaling are reported in the red boxes in figure. Thin green arrows indicate activation, while red T arrows indicate direct inhibition. Thick green arrows indicate inhibitory phosphorylation. (p) Indicates a phosphate group. Abbreviations: IRS1/2, insulin receptor substrate 1/2; PIP3, phosphatidylinositol 3,4,5-triphoshate; PIP2, phosphatidylinositol 4,5-bisphoshate; PDK1, phosphoinositide-dependent kinase 1; mTORC2, mammalian target of rapamycin complex 2; FOXO1, forkhead box protein O1; GSK3, glycogen synthase kinase 3; GS, glycogen synthase; AS160, AKT substrate of 160 kDa; GLUT4, glucose transporter 4; Grb2, growth factor receptor-bound protein 2; SOS, son of sevenless; KRAS, Kirsten rat sarcoma viral oncogene homolog; RAF, RAF proto-oncogene serine/threonine-protein kinase; MEK, mitogen-activated protein kinase kinase; ERK, extracellular signal-regulated kinase.

**Figure 2:**
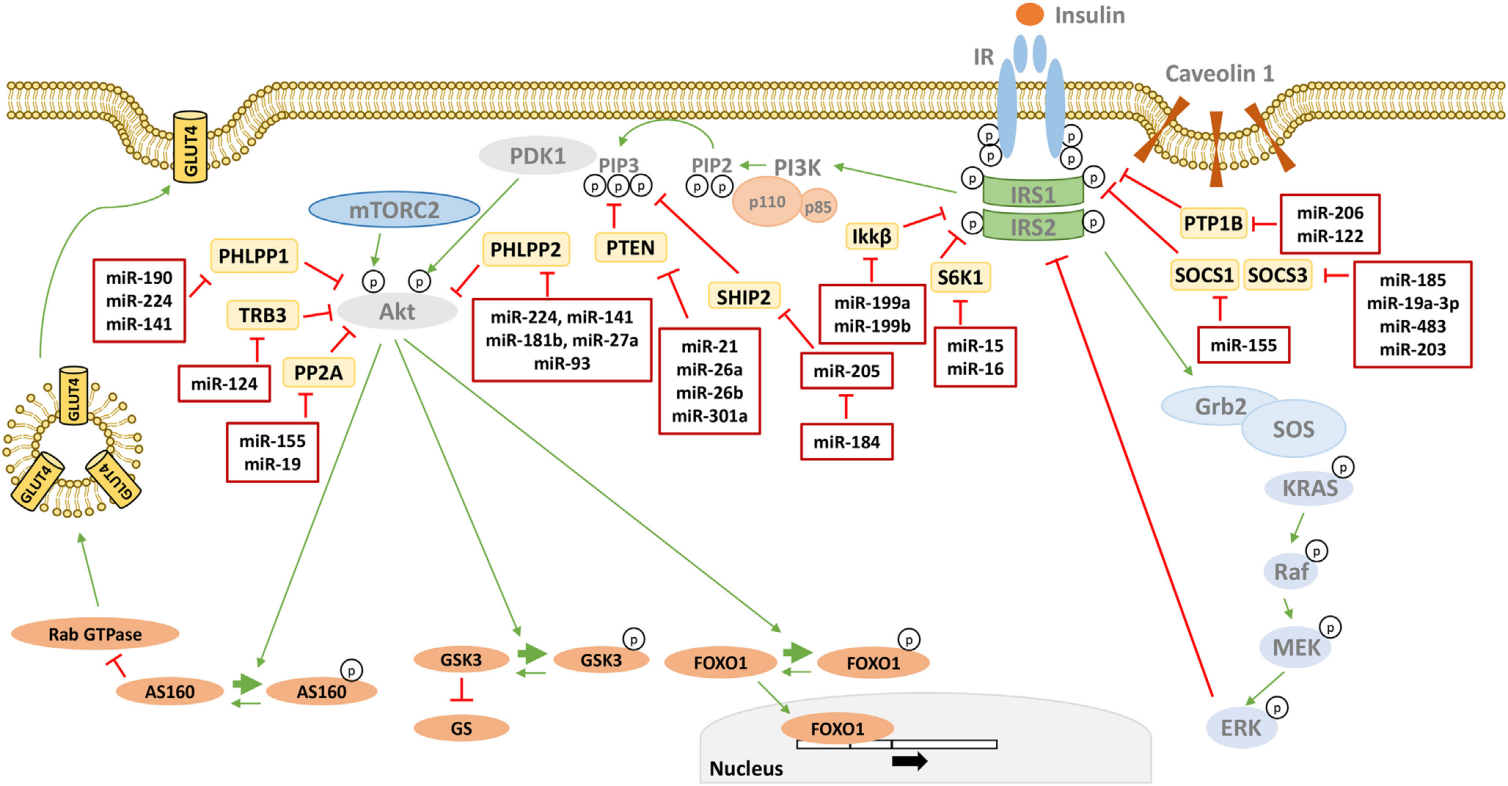
MicroRNAs (miRNAs) targeting phosphatases of insulin signaling. Many phosphatases have a negative effect on insulin signal transduction by targeting critical mediators. miRNAs directly targeting these phosphatases are reported in the red boxes in figure. Thin green arrows indicate activation, while red T arrows indicate direct inhibition. Thick green arrows indicate inhibitory phosphorylation. (p) Indicates a phosphate group. Abbreviations: PTP1B, protein tyrosine phosphatase 1B; SOCS1/3, suppressor of cytokine signaling 1/3; S6K1, Ribosomal protein S6 kinase beta 1 IKKβ, inhibitor of nuclear factor kappa-B kinase subunit beta; SHIP2, SH2-containing 5′-inositol phosphatase; PTEN, phosphatase and tensin homolog; PHLPP1/2, PH domain and leucine-rich repeat protein phosphatases 1/2; TRB3, tribbles homolog 3; PP2A, protein phosphatase 2.

Furthermore, determining whether and how changes in miRNA levels have a causative effect in the onset of insulin resistance is also under investigation. Several studies have identified miRNAs as regulators of key components of the insulin signaling, contributing to their downregulation in insulin-resistant conditions, and they are shown in Figure 1 and Table 1. However, well-known molecular mechanisms, able to negatively regulate the critical nodes of the insulin signaling, may also be modulated by miRNAs. Hence, miRNAs may also affect indirectly the activity of these nodes through their negative regulators. Examples are shown in Figure 2 and are made explicit in the text below.

**Table 1:** MicroRNAs (miRNAs) involved in insulin signaling.

Target	miRNA	Cell type/tissue	Reference
Insulin receptor	miR-15	Hepatocytes	(39)
	bmiR-195	HepG2	(40)
	miR-128a	Skeletal muscle, breast	(41, 42)
	miR-144	Blood	(43)
	miR-135	C2C12	(44)
	Let-7	C2C12	(45)
	miR-96	Hepatocytes	(46)

Caveolin-1	miR-103/107	SGC7901, liver, adipose tissue	(47, 48)
	miR-124	N2A/APP695swe	(49)

Insulin receptor substrate 1 (IRS-1)	miR-128a	Skeletal Muscle	(41)
	miR-144	Blood	(43)
	Let-7, lin-28	C2C12	(45)
	miR-126	Endothelial cells	(50)
	miR-23a	NSCLC	(51)
	miR-29	Myocytes, Skeletal muscle	(52, 53)
	miR-145	HepG2	(54, 55)
	miR-96	Hepatocytes	(47)

IRS-2	miR-135a	Skeletal muscle	(56)
	miR-126	β-cells	(57)
	miR-33a/b	Hepatocytes	(58)

PDK1	miR-375	β-cells	(59)
	miR-210	Endothelial cells	(60)

Phosphatidylinositol 3-kinase	miR-128a	Skeletal muscle	(41)
	Let-7	HepG2	(45)
	miR-126	Endothelial cells	(57)
	miR-503	NSCLC	(61)
	miR-29	Skeletal muscle	(53)
	miR-320	Adipocytes	(62)
	miR-378	Hepatocytes	(63)

AKT	miR-128a/b	Skeletal muscle	(41)
	miR-145	HepG2	(55)
	miR-126	β-cells	(57)
	miR-143	Liver	(64)
	miR-1	H9C2	(65)
	miR-423	Hepatocytes	(66)
	miR-29	Adipocytes	(67)
	miR-26b	Adipocytes	(68)

### Insulin Receptor/Insulin Receptor Substrate

The first critical node in the insulin signaling is represented by the interaction between IR and IRS, which share a common mechanism of activation based on tyrosine phosphorylation. In addition to decreased levels of both IR (69) and IRS, protein tyrosine phosphatases (PTPases) are able to dephosphorylate tyrosine residues on the IRβ subunit and IRS docking molecules, resulting in a dampening of the insulin signal transduction. Therefore, an abnormal overexpression of the PTPases contributes to the insulin resistance in diabetic states in both rodents and humans (70, 71). Several PTPases are currently attractive therapeutic targets for obesity and T2D, including PTP1B (72, 73). Indeed, PTP1B is widely expressed and detectable in the insulin-responsive tissues. In this respect, in high-fat diet (HFD) fed mice or hepatocyte models with insulin resistance, the expression of miR-122 is downregulated as a consequence of the HNF4α phosphorylation by c-Jun N-terminal kinase 1 (JNK1). This event leads to hepatic insulin resistance through the PTP1B induction and may be overcome by the chemical inhibition of JNK and, subsequently, the rescue of the miR-122/PTP1B levels (74). Moreover, the increase in miR-206 expression is responsible for the downregulation of PTP1B with an improvement of the insulin signaling, thus protecting glomerular podocytes against fructose-induced injury and the consequent proteinuria (75).

In addition to being adaptor proteins that act as negative regulators of cytokine and growth factor signaling, the proteins of the suppressor of cytokine signaling (SOCS), in particular SOCS1 and SOCS3, negatively regulate the insulin signaling, linking the cytokine signaling to insulin resistance (76). The SOCS proteins are of particular importance because they are upregulated in states of insulin resistance, such as obesity, and contribute to the insulin resistance through the inhibition of the tyrosine phosphorylation of IRS (77, 78). Found to be significantly downregulated in diabetic patients and mouse models, the miR-185 levels are directly correlated with blood glucose concentration and inversely correlated with the SOCS3 levels in diabetes patients. Therefore, the restoration of miR-185 expression may serve as a potentially promising and efficient therapeutic approach for diabetes (79). The levels of miR-19a-3p are also significantly decreased in diabetic patients and inversely correlated with the SOCS3 levels, suggesting that the downregulation of miR-19a-3p may contribute to the dysfunction of pancreatic β cells through SOCS3. Hence, the miR-19a-3p/SOCS3 axis may also become a potential therapeutic target for diabetes (80). Additionally, miR-483 affects the insulin transcription and secretion by targeting SOCS3 in pancreatic β cells and an imbalance of miR-483 and SOCS3 may play a crucial role in the pathogenesis of T2D (81). Furthermore, the accumulation of the pro-inflammatory M1 macrophages, which occurs in the adipose tissue, is a central event leading to the metabolic complications related to obesity. The M1 macrophage polarization is due to a significant decrease in the protein levels of SOCS1, a proven target of miR-155, followed by the activation of STAT1 and suppression of STAT6. As miR-155 has been observed to be upregulated in both adipocyte-derived micro-vesicles from obese mice and in macrophages treated with those micro-vesicles, it may mediate the obesity-induced imbalance in the M1-to-M2 macrophage ratio in adipose tissue, contributing to the chronic inflammation state and local insulin resistance (82). Finally, the chronic *Helicobacter pylori* infection is associated with an increased risk of developing T2D, as it induces hepatic insulin resistance by the c-Jun/miR-203/SOCS3 axis (83).

The serine–threonine phosphorylation of IR and IRS docking proteins is a major mechanism for the negative modulation of the insulin signaling. Indeed, the serine phosphorylation diminishes the IR tyrosine kinase activity and decreases the IR–IRS coupling. There are also consensus sequences in IRS that make it susceptible to a wide variety of serine–threonine kinases, including protein kinase C (PKC), extracellular signal-regulated kinase (ERK), mammalian target for rapamycin (mTOR)/Ribosomal protein S6 kinase beta 1 (S6K1), JNK, and inhibitor of κB kinase beta (IKKβ) (84). The ERK and S6K1 are activated by insulin, indicating that the serine phosphorylation of IRS represents a negative feedback mechanism for the insulin signaling pathway, and thus these kinases cause a desensitization to the insulin action under conditions of nutrient excess, inflammation, and cell stress responses. JNK and IKKβ are activated by inflammatory stimuli, such as tumor necrosis factor alpha (TNF-α), contributing to insulin resistance in obesity, while conventional and novel PKC isoforms are activated *via* their recruitment to the plasma membrane by diacylglycerol, accumulated into the cells as a consequence of the increased availability of free fatty acids. miR-15 and miR-16 belong to a common precursor family and are highly conserved. The deletion or downregulation of these two miRNAs has been shown to accelerate cell division by modulating the expression of genes involved in the control of the cell cycle. An in-depth study performed in MDA-MB-231 breast cancer cells has demonstrated that both miR-15 and miR-16 target Ribosomal protein S6 kinase beta 1, control cell proliferation, and cause apoptosis (85). In insulin-resistant conditions, a posttranscriptional regulation of Ribosomal protein S6 kinase beta 1 may be undertaken by miR-15 and miR-16 or by other miRNAs yet to be identified. Furthermore, both miR-199a and miR-199b negatively regulate IKKβ by binding its 3′-UTR, a fact that is supported by the following studies. Cancer progression is an abnormal form of tissue repair (86), characterized by chronic inflammation in which IKKβ has a critical role. The differential expression of IKKβ in epithelial cells isolated from patients with ovarian cancer is inversely correlated with the levels of miR-199a (87). In addition, the inflammatory response plays an important part in the progression of spinal cord injury and the downregulation of miR-199b promotes this process through the IKKβ–NFκB pathway and microglial cell activation (88). Additionally, miR-377 and let-7a directly interact with ERK2 and alter its expression, significantly reducing the basal trophoblast proliferation in placenta (89). Moreover, it has been demonstrated that miR-216b exerts its tumor suppressor function through the inhibition of the Kirsten rat sarcoma viral oncogene homolog (KRAS)–ERK pathway. Indeed, the miR-216b levels inversely correlate with the protein levels of KRAS during nasopharyngeal tumorigenesis and a decrease in its expression is related to the advanced clinical stage and lymph node metastasis *via* the upregulation of KRAS (90). The latter has also been identified as a target of miR-134 in renal cell carcinoma. Thus, miR-134 may act as a tumor suppressor in cell proliferation and epithelial–mesenchymal transition through the KRAS/ERK pathway (91). In HepG2 and MHCC97L cancer cells, miR-30a overexpression completely blocks the activation of the KRAS/ERK pathway by directly binding the 3′-UTR of KRAS. Overall, these findings indicate that miR-30a may be involved in the cell growth, apoptosis, and metastasis of hepatocellular carcinoma cells (92). Recently, we have demonstrated that miR-190a downregulation plays a role in the methylglyoxal (MGO)-induced insulin resistance in endothelial cells by increasing the KRAS protein levels. This effect attributed to miR-190a may explain the ERK1/2 hyper-activation and IRS-1 phosphorylation on Ser-616, which we had previously observed in the presence of MGO both *in vitro* and *in vivo* (93). Thus, our study highlights miR-190a as a new candidate for the identification of strategies aimed at ameliorating the vascular function in diabetes (94).

### Phosphatidylinositol 3-Kinase

The increased production of phosphatidylinositol (3,4,5)-trisphosphate [PtdIns (3,4,5) P3], abbreviated PIP3, the lipid second messenger molecule generated through the phosphorylation of phosphatidylinositol (4,5)-bisphosphate (PIP2) by PI3K, is a decisive event in the insulin signaling related to metabolism (95). Indeed, its binding to proteins with a PH domain, such as the phosphoinositide-dependent kinase 1 (PDK1), is responsible for the activation of the serine/threonine kinase AKT/PKB and atypical PKCs (αPKCs). Along with the AKT/PKB activation, αPKCs represent important downstream effectors of PI3K in mediating the metabolic effects of insulin, as they are able to affect the translocation of glucose transporter 4 (GLUT4) and the insulin-induced glucose uptake in the adipocytes and muscles. A decreased activation of αPKCs has been reported in the muscles of T2D humans and rodents, whereas the AKT/PKB activation may result to be unchanged (96).

In addition to the inhibition due to the regulatory subunit of PI3K (p85), PIP3 produced by this enzyme can be hydrolyzed by phospholipid phosphatases, such as phosphatase and tensin homolog (PTEN) deleted on chromosome 10 and SH2-containing 5′-inositol phosphatase-2 (SHIP2), which dephosphorylate and inactivate PIP3 on the 3′- or 5′-position of the inositol ring, respectively. Therefore, PTEN and SHIP2 can exert negative regulatory influences on the insulin signaling, causing PDK1 to cease its activity (97). In hepatocytes, unsaturated fatty acids upregulate the expression of miR-21 and, subsequently, inhibit the tumor suppressor PTEN, since its deletion in the liver leads to insulin resistance, steatosis, inflammation, and cancer. Consistent with these data, the miR-21 expression has been found to be increased in the liver of HFD rats and in obese patients, together with a diminished expression of PTEN and steatosis (98). In addition, miR-21 levels are significantly downregulated in insulin-resistant adipocytes. Considering that PTEN is a well-known target of miR-21, Ling et al. (99) have overexpressed miR-21 in 3T3-L1 adipocytes with insulin resistance induced by high glucose and high insulin to demonstrate that miR-21 may contribute to insulin resistance or diabetes. In more detail, in the presence of high levels of miR-21, a relevant increase in the insulin-induced phosphorylation of AKT (Ser-473), the translocation of GLUT4 to the plasma membrane and the insulin-induced glucose uptake have been observed in concomitance with the decrease in the protein levels of PTEN, suggesting this miRNA as a new therapeutic target for metabolic diseases, such as T2D and obesity (99). Furthermore, the decreased miR-26b expression observed in visceral adipose tissue of obese rodent models and human patients may also be involved in obesity-related insulin resistance by interrupting the PTEN/PI3K/AKT pathway. Indeed, miR-26b modulates the insulin-stimulated AKT activation *via* the inhibition of its target gene PTEN, promoting the GLUT4 translocation to the plasma membrane and insulin-induced glucose uptake in human mature adipocytes. Moreover, the expression of miR-26a is dramatically downregulated in the serum and islets of both HFD and db/db mouse models, while its overexpression protects against HFD-induced diabetes and maintains prolonged normal-glycemic time in HFD mice through an improvement of the pancreatic β cell function (100). Finally, IL-6 has been implicated in the pathogenesis of insulin resistance. *In vivo* and *in vitro* treatments with IL-6 cause the downregulation of miR-301a in the liver, accompanied by an impairment of the AKT/glycogen synthase kinase (GSK) pathway and glycogenesis. The contribution of miR-301a to the IL-6-induced insulin resistance has been demonstrated to be due to a direct effect on the PTEN expression (101).

The lipid phosphatase SHIP2 is a target of miR-205 in epithelial cells. In addition, the corneal epithelial-specific miR-184 can interfere with the ability of miR-205 to suppress the SHIP2 levels, giving an example of a miRNA that negatively regulates another to maintain the levels of a target protein. Interfering with the miR-205 function, miR-184 leads to a dampening of the AKT activity *via* the SHIP2 induction. This is associated with a marked increase in the keratinocyte apoptosis and cell death (102). As aggressive squamous cell carcinoma cells exhibit elevated levels of miR-205, the blockage of the miR-205 activity with its specific antagomiR- or *via* the ectopic expression of miR-184 may be novel therapeutic approaches for its treatment (103).

### AKT/PKB

The serine/threonine-specific protein kinase AKT/PKB mediates most of the metabolic actions of insulin through the phosphorylation of several substrates, including other kinases, signaling proteins, and transcription factors. The AKT/PKB activity is regulated by several inhibitory molecular mechanisms, including tyrosine phosphatases and adaptor proteins.

Protein phosphatase 2A (PP2A), which accounts for ~80% of the serine/threonine phosphatase activity in the cells, regulates the activities of many protein kinases involved in the insulin action, including AKT/PKB. Several studies indicate that PP2A is hyper-activated in diabetic states (104). In many cancers, the loss of the PP2A activity has been associated with tumorigenesis and drug resistance, due to the failure of turning off survival signals, such as those involving AKT/PKB (105). In addition, recent studies have demonstrated that a number of clinically significant miRNAs, including miR-19 (106) and miR-155 (107) may have PP2A as a target.

Other serine/threonine phosphatases have been implicated in the insulin actions. Protein phosphatases 2B, also known as calcineurin, has been shown to dephosphorylate AKT/PKB. In addition, two novel members of the PP2C family involved in regulation of the insulin signaling are the PH domain leucine-rich repeat protein phosphatases 1 and 2 (PHLPP-1 and PHLPP-2), which dephosphorylate both AKT/PKB and PKCs (108). The overexpression of PHLPP1 in cells impairs AKT/PKB and GSK3 activity, resulting in a decrease of the glycogen synthesis and glucose transport (109). Elevated levels of PHLPP1 have been found in the adipose tissue and skeletal muscle of obese and/or diabetic patients and correlate with decreased AKT2 phosphorylation levels (109, 110). Regarding the regulation of PHLPP1 expression by miRNAs, published studies have revealed that miR-190 is responsible for the downregulation of PHLPP1, contributing to the AKT/PKB activation and carcinogenesis in human bronchial epithelial cells (111, 112). Moreover, several other miRNAs affect the PHLPP1 levels in cancer cells while there is no information available on a potential regulation of PHLPP1 at the posttranscriptional level in insulin-resistant conditions. On the contrary, miRNA-181b is able to improve the glucose homeostasis and insulin sensitivity by modulating the PHLPP2 levels in the endothelial cells and, thus, affecting the endothelial function in the white adipose tissue (113). miRNA-181 has been demonstrated to target PHLPP2 in additional conditions, such as in fibrous overgrowths induced by cutaneous injury, called keloid (114) and in luminal breast cancer cells (115). Among the other miRNA identified as regulators of PHLPP2, it is worth mentioning miRNA-27a in gastric cancer cells (116) and miR-93 in glioma cell lines and clinical glioma tissues (117). Indeed, the former is highly expressed in the omentum of the obese patients (118) and represses adipocyte differentiation (119), while the latter is overexpressed in adipose tissue of polycystic ovary syndrome patients and women with insulin resistance and regulates the GLUT4 levels (120). Hence, in these conditions with altered miRNA-27a and miR-93, PHLPP2 expression may also be affected. However, this hypothesis remains to be demonstrated. Finally, cases of miRNAs able to modulate both PHLPP1 and PHLPP2 have been reported in the literature, including miR-224 in esophageal squamous cell carcinoma (121) and in colorectal cancer (122), and miR-141 in non-small cell lung cancer (123).

Tribbles homolog 3 (Trb3) is a member of the family of pseudo-kinases and is thought to function as an adaptor protein. Indeed, binding to the un-phosphorylated AKT/PKB and blocking its phosphorylation and activation *in vivo*, it is another brake on the insulin signaling. The Trb3 expression is induced in the liver (124) as well as in the sheletal muscle (125), in fasting and diabetic conditions. In non-alcoholic fatty liver disease and related metabolic diseases, an increase in hepatic triglyceride contents usually occurs and it may be promoted by miR-124 through its confirmed target, TRB3. Indeed, this miRNA is upregulated in the liver of C57BL/6 mice fed a short-term HFD, which leads to an increase in the accumulation of triglycerides and in the expression of lipogenic genes in the liver, together with an enhancement in the AKT/PKB activity (126).

## The Role of miRNAs in the Cellular Destiny of Glucose

**Figure 3:**
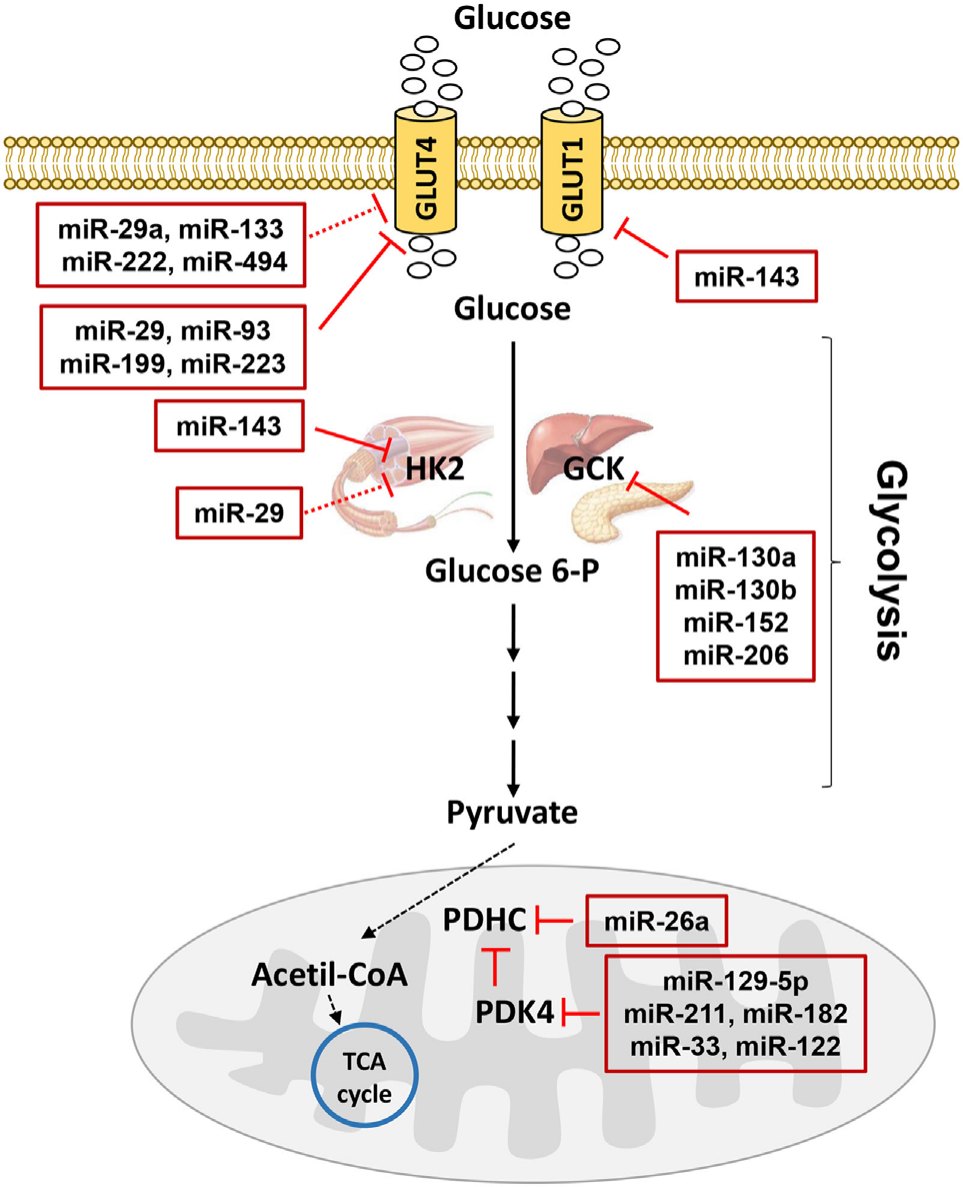
MicroRNAs (miRNAs) involved in glucose metabolism. Glucose transporters (GLUTs) mediate glucose entrance into the cells where it is transformed in pyruvate by glycolysis. Hexokinase 2 (HK2) catalyzes the conversion of glucose to glucose 6-phosphate in muscle, while glucokinase catalyzes the same reaction in liver and pancreas. In mitochondria, pyruvate dehydrogenase complex (PDHC) transforms pyruvate in acetil-coenzyme A (CoA) allowing the entrance to the tricarboxylic acid (TCA) cycle. PDHC activity is controlled by pyruvate dehydrogenase kinase 4 (PDK4). miRNAs targeting GLUTs and enzymes catalyzing glucose metabolism are reported in the red boxes in figure. Red T arrows indicate direct inhibition, while red T dotted arrows indicate indirect inhibition.

Insulin resistance is a common feature in patients with T2D and it precedes the development of hyperglycemia. Nevertheless, over the last few decades, despite extensive efforts aimed at gaining a better understanding of the insulin signaling, researchers have been unable to determine many causes of the disease. Therefore, to identify other factors with additive, synergistic or antagonistic effects on the glucose homeostasis, it is important to carefully consider also the cellular metabolic destiny of glucose. Much previous work has been focused on the activity of rate-limiting enzymes in metabolic pathways, regulated by either substrate/metabolite availability or gene expression (127–129). However, a single enzyme strategy has often been proved to be ineffective because a coordinated dysregulation of several pathways or several components of a single main pathway may occur, due to alterations in the expression of transcription factors and co-factors. In addition, the regulation at the posttranscriptional level by miRNAs with possible simultaneous alterations in multiple steps may also have pathophysiological implications and, thus, it is imperative to reveal the mechanisms based on the miRNA action. Indeed, only a few studies have been published demonstrating a direct regulation of metabolic enzymes due to miRNAs (Figure 3) or an indirect one through the modulation of transcription factors by miRNAs. Therefore, progress in the identification of the miRNAs involved in the pathways related to the glucose metabolism is essential and it may suggest new targets to use for therapy.

### The Glucose Uptake

Previous studies have indicated that glucose transport is a rate-limiting step for the glucose metabolism. Indeed, due to its hydrophilic nature, glucose cannot penetrate the lipid bilayer and therefore specific transporter proteins, known as GLUTs, are required to facilitate the diffusion into cells down its concentration gradient. GLUT1 is a widely expressed isoform that provides glucose transport under basal conditions for many cell types. Moreover, the exposure of cells to several stresses results in an acute increase in the entry of glucose into the cells by GLUT1 (130), considered as an adaptive response to meet specific energy demands. The activity of GLUT1 is modulated through changes in its gene expression also due to miRNAs, such as miR-143 in the placenta of obese women with gestational diabetes mellitus (131) and many others altered in cancer (132, 133). GLUT4 is the major mediator of extracellular glucose clearance, playing a key role in the regulation of glycemic homeostasis. Indeed, in the skeletal muscle and adipose tissue, GLUT4 mediates the insulin-stimulated glucose uptake by a mechanism involving the translocation between cellular compartments and the increased glucose entry rate is due to a greater amount of GLUT4 at the cell membrane under insulin stimulation (134). Due to a reduction of the GLUT4 expression and/or translocation, the decreased insulin-stimulated glucose uptake is a common feature and contributes to the impairment in the glycemic control in the adipose tissue and skeletal muscle of pre-diabetic and diabetic subjects (135–138). Several miRNAs, many of which not yet identified, are involved in the expression of GLUT4 or proteins responsible for the GLUT4 translocation. As the levels of these miRNAs may be altered in metabolic diseases, their contribution to insulin resistance and diabetes has been suggested. In conditions related to insulin resistance and T2D, the role of miRNAs on the GLUT4 expression has been investigated only in a few studies performed not only in the skeletal muscle but also in adipose and cardiac tissues or cells. miR-29 (53), miR-93 (120), miR-199a (139), and miR-223 (140, 141) have a direct effect on the GLUT4 mRNA levels. Moreover, other miRNAs have an effect on regulators of the GLUT4 expression. For instance, in addition to its action at the 3′UTR of GLUT4, miR-29a targets peroxisome proliferator-activated receptor delta, which in turn reduces a transcriptional coactivator essential for GLUT4, namely peroxisome proliferator-activated receptor gamma coactivator 1 alpha (142). Additionally, miR-133 targets Krueppel-like factor 15 in the cardiomyocytes (143), while miR-222 targets estrogen receptor alpha in the omental adipose tissues from gestational diabetes mellitus subjects (144). Furthermore, only a few miRNAs have been linked to the expression of proteins involved in the GLUT4 translocation machinery in the muscle, impacting on the glucose uptake. One such miRNA is miR-494, which regulates syntaxin-binding protein 5 in C2C12 cells (145). All these miRNAs related to GLUT4 represent promising targets for preventive and/or therapeutic approaches aimed at improving glycemic control and, therefore, deserve future investigation.

### The Glycolytic Metabolism

Glycolysis is the pathway through which glucose is broken down into pyruvate, providing the substrate for energy production as well as for energy storage through glycogenesis and lipogenesis. Immediately following its transport into cells, hexokinases phosphorylate the glucose to trap it within the cell. Hexokinase 2 (HK2) expression and activity are reduced in the skeletal muscle of subjects with T2D (146). As insulin induces the HK2 expression in the skeletal muscle *via* PI3K and the p85α regulatory subunit of PI3K is a validated target of miR-29 (147), this miRNA has been suggested being an indirect cause of the reduction in the HK2 mRNA levels with a consequent loss of its activity observed in skeletal muscle cells overexpressing miR-29 (53). Conversely, HK2 is highly expressed in tumors (148, 149) and its regulation by miRNAs has been demonstrated in several studies. Since miR-143 has been found to be downregulated in cancer cells and since it is able to directly target HK2 (150–152), its upregulation observed in diabetic conditions (64, 131) may explain the reduced expression of HK2. However, this hypothesis remains to be demonstrated.

The glucokinase (GCK, the predominant hexokinase isoenzyme in the hepatic and pancreatic β cells) was one of the first candidate genes to be identified as a human “diabetogene,” since its heterozygote inactivating mutation was reported to be a sub-type of the maturity-onset diabetes of the young, causing reduced insulin secretion and hyperglycemia. In the liver, the status of GCK as a prominent player in the regulation of glucose metabolism supports the important role of the glycolytic process in the regulation of the hepatic glucose production and, consequently, in the maintenance of glucose homeostasis (153). The transcriptional regulation of GCK has been extensively studied (154) and a dramatic induction by insulin has been demonstrated, whereas miRNAs involved in its posttranscriptional regulation are still “in the black box.” Recently, GCK protein levels have been found to be downregulated in the Goto-Kakizaki rat islets, in accordance with the elevated expression of miR-130a, miR-130b, and miR-152 (155). In addition, a novel mechanism of posttranscriptional regulation of GCK by miR-206 has been revealed (156). As an induction in the expression of GCK promotes hepatic glucose utilization, the development of the GCK activators has generated exciting results at the pre-clinical phase in the diabetic field, but has also revealed some limitations (157). On the other hand, the identification of miRNAs as modulators of the GCK levels may represent an alternative approach and show greater promise from a therapeutic perspective.

In adipocytes, the glucose phosphorylation catalyzed by hexokinase is not a rate-limiting step, while 6-phosphofructo-2-kinase/fructose 2,6-bisphosphatase-3 (PFK-2/FBPase-2) is more significant as it serves as a coordinator that links adipocyte glycolysis, glycolysis-driven lipogenesis, and the inflammatory response. The latter enzyme is downregulated by miR-26b in osteosarcoma cells (158). In addition, the miRNA-26 family is required for human adipogenesis (159) and miR-26b modulates insulin sensitivity in adipocytes by interrupting the PTEN/PI3K/AKT pathway (68). Thus, this miRNA may also play a yet undiscovered role in targeting PFK-2/FBPase-2 in adipocytes.

### The Mitochondrial Oxidative Metabolism

Pyruvate is a key intermediate in several metabolic pathways. Made from glucose through glycolysis, pyruvate molecules can be converted back into carbohydrates (such as glucose) *via* gluconeogenesis or into fatty acids through a reaction with acetyl-coenzyme A (CoA). In addition, pyruvate supplies energy to cells through the tricarboxylic acid (TCA) cycle (also known as the citric acid cycle or the Krebs cycle) in aerobic conditions or, alternatively, ferments to produce lactate in anaerobic conditions. Hence, a stringent control of the destiny of pyruvate is critical for cellular homeostasis and the following enzymes in its metabolism are of great importance:

(a) Mitochondrial pyruvate carrier (MPC), which is located in the mitochondrial inner membrane and carries pyruvate from the intermembrane space into the mitochondrial matrix space. Recently, MPC activity has been shown to be activated by the mitochondrial deacetylase sirtuin-3 (160). As yet, no miRNAs have been identified as regulators of MPC.
(b) Pyruvate carboxylase (PC), which converts pyruvate into oxaloacetate necessary for lipogenesis in the fed state and for hepatic gluconeogenesis in the fasted state. Since PC catalyzes the committed step in gluconeogenesis and the PC protein levels correlate with glycemic levels in subjects with T2D, factors that may affect its expression and/or activity are receiving growing interest within the scientific community, in accordance with the increased standing of PC as a potential therapeutic target (161). Among these factors, miRNAs may be included. As no research has been undertaken to date, future studies are required to find a possible involvement of miRNAs in the PC regulation.
(c) Pyruvate dehydrogenase complex (PDHC), which inserts pyruvate into the TCA cycle catalyzing the irreversible decarboxylation of pyruvate into acetyl-CoA. Interconnecting glycolysis and the TCA cycle, PDHC is critical for the control of the glucose metabolism in the fasted-fed cycle. Indeed, PDHC is highly active in the fed state and glucose is oxidized to generate energy or is rerouted to glycogen as an energy storage. By contrast, this enzyme is inactivated in the fasted state, when glucose is in demand and three carbon compounds need to be conserved for the hepatic gluconeogenesis to maintain the whole-body glucose homeostasis. In this condition, lipolysis is activated and fatty acids become the preferred fuel (the glucose-fatty acid cycle) (162). Hence, considering the key role it plays in glucose disposal, a close control of the PDHC expression and activity is required. Indeed, an inappropriate suppression of its activity in the skeletal muscle promotes the development of hyperglycemia through excessive gluconeogenesis.

Regarding the posttranscriptional regulation of PDHC expression, miR-26a regulates the glucose metabolism of colorectal cancer cells by directly targeting the E3 binding protein subunit, also known as component X (PDHX) of PDHC, efficiently improving the accumulation of pyruvate and decreasing the production of acetyl-CoA (163). However, in addition to its metabolic role in tumorigenesis targeting PDHX, Fu et al. (164) have demonstrated that miR-26a regulates the insulin signaling and metabolism in the liver. In addition, overweight individuals show a decrease in the miR-26a hepatic expression compared to lean controls and a downregulation has been observed also in two different mouse models of obesity (164). On the other hand, the expression of miR-26a is rapidly induced in response to high glucose in the endothelial cells and it may be an important regulator in the progression of skin wounds of diabetic mice, being able to affect the angiogenic response after injury (165). Questions about a potential role of miR-26a in metabolic diseases through its effect on PDHX and about the existence of other miRNAs able to regulate PDHC require further investigation.

The control of PDHC activity is achieved through a reversible, inactivating phosphorylation by pyruvate dehydrogenase kinase (PDK) (166). Of all the known isozymes, pyruvate dehydrogenase kinase 2 and pyruvate dehydrogenase kinase 4 are the most widely distributed and are highly expressed in the heart, liver, and kidneys in humans and rodents. PDK4 is also abundant in the skeletal muscle, which shows high glucose utilization and fatty acid oxidation rates. Not only can the PDK4 activity be regulated by metabolites but also the PDK4 expression is influenced by many factors that have been extensively studied (167). At the transcription level, forkhead box protein O1 (FOXO1) has been proposed as one of the main regulators of the PDK4 expression, mediating the insulin-dependent repression as a consequence of the AKT/PKB-dependent phosphorylation and nuclear exclusion. Hence, under insulin stimulation and PDK4 repression, the skeletal muscle is able to switch from lipid oxidation to elevated glucose uptake, oxidation, and storage since PDHC is in the active state. During short-term energy deprivation, the dephosphorylated FOXO1 remains in the nucleus and binds the PDK4 promoter, inducing its expression. Fatty acid oxidation is then preferred to glucose oxidation, as a consequence of the PDHC inhibition (168).

In obese and diabetic subjects, insulin resistance is mostly characterized by a reduction in both the stimulation of the glucose metabolism and the suppression of lipid utilization in the skeletal muscle. Thus, the capacity to switch between fuels is defective, a condition known as “metabolic inflexibility” (a failure to adapt the metabolism to the fasted-to-fed state transition) (169). Since the incapacity of insulin to downregulate PDK4 has been demonstrated in obese and diabetic subjects (170), the consequent increase in the PDK4 levels associated with an inappropriate suppression of the PDHC activity may promote the development of metabolic diseases (171).

Furthermore, the environment may also have an effect on the PDK4 expression and glucose metabolism. Indeed, changes in the PDK4 mRNA expression in the skeletal muscle have been shown to correlate with the improvement in insulin sensitivity in obese patients after bariatric surgery, demonstrating the link between PDK4 expression and fat mass (172). In this regard, in the skeletal muscle, an inverse correlation has been demonstrated between the PDK4 expression and DNA methylation, involving specifically cytosines in the +160 and +446 regions of the PDK4 promoter. Obesity is associated with an altered methylation and expression of PDK4, while weight loss by gastric bypass surgery is able to normalize the levels of both PDK4 methylation and PDK4 expression to those observed in normal-weight, healthy controls (173). The methylation status and expression levels of PDK4 are also altered in T2D patients, but exercise is able to revert these effects (174). Therefore, changes in the environment, such as weight loss and exercise, have significant beneficial outcomes also by modulating the PDK4 expression.

As an increase in the PDHC activity by a PDK4 inhibitor promotes glucose oxidation and lowers the blood glucose concentration, small molecule inhibitors for PDKs are promising therapeutic agents for patients with metabolic diseases. Actually, therapeutic interventions have already been tested to reduce the PDK4 expression and promote glucose disposal in animal models of diabetes. Preliminary studies have shown encouraging results with the oral administration of dichloroacetate (175), but this compound has a low potency and specificity for PDK4 and is toxic (176, 177). The modulation of the PDK4 expression by miRNAs may be an alternative therapeutic approach. However, at present, there are only a few very recent studies regarding the posttranscriptional regulation of PDK4 by miRNAs. Most of these have been performed using cancer cells, in the wake of the mounting interest in the reprogramming of the glucose metabolism in cancer. In relation to miR-129-5p, it targets PDK4 and inhibits glycolysis, thus retarding tumor growth and impairing carcinogenesis in the liver and colonization in the lung (178). The tumor suppressor miR-211 is significantly reduced or absent in non-pigmented melanoma cells and is lost during human melanoma progression. This event represents an important metabolic switch through the regulation of PDK4 (35). Moreover, miR-33 and miR-122, which are commonly altered in metabolic disorders, have been demonstrated to directly target PDK4 in the macrophages to promote cholesterol efflux and reduce atherosclerosis (179) and in the CD133 (+) hepatocellular cancer stem cells (180), respectively. Furthermore, miR-182 is able to modulate glucose utilization by targeting PDK4 both in the muscle of short-term HFD mice (181) and lung cancer cells (35), providing confidence of its effectiveness in other similar examples.

## Conclusion

MicroRNAs have emerged as important regulators of multiple aspects of the metabolic homeostasis and changes in their expression may prove to be crucial in determining the metabolic status, normal or pathological. In general, miRNAs are able to regulate the cellular metabolism in two different ways. Indeed, miRNAs modulate transcription factor or signaling protein expression, which in turn affects the levels and/or the activity of the metabolic enzymes. Alternatively, miRNAs control the production of certain metabolites by directly regulating the expression of those genes encoding metabolic enzymes at the posttranscriptional level.

In the last few years, much work has been carried out taking into account the metabolic reprogramming of transformed cells (the Warburg effect), identifying miRNAs able to switch from the TCA cycle to aerobic glycolysis (lactate production) by targeting metabolic transporters (like GLUTs) or enzymes (HK2 and PDKs) and by constitutively activating, among others, the PI3K/AKT/mTOR pathway. Paradoxically, the role of miRNAs in metabolic diseases still remains not well explained and further investigation is required, supported by the increasing evidence of the association between miRNAs and the cancer metabolic reprogramming (182). In addition, most researchers seem to focus on defects in the insulin signaling rather than on other aspects of the metabolism, but it would be appropriate to consider the metabolism in a broader perspective, since miRNAs are involved in several, if not all, metabolic processes.

In relation to T2D, the discovery of an association between miRNAs and metabolism may attract significant interest not only from a scientific but also from a clinical point of view, providing an appealing therapeutic approach, owing to the small size of miRNAs and specificity on their targeting gene, and suggesting them as candidate molecular biomarkers for the diagnosis and/or prognosis of T2D in the future. Additionally, in combination with inhibitors highly specific for metabolic enzymes, miRNAs may constitute a powerful strategy to win the battle against T2D.

## Author Contributions

All authors listed have made a substantial, direct, and intellectual contribution to the work and approved it for publication.

## Conflict of Interest Statement

The authors declare that the research was conducted in the absence of any commercial or financial relationships that could be construed as a potential conflict of interest. The reviewer RI and handling editor declared their shared affiliation.
